# Does cooperation mean kinship between spatially discrete ant nests?

**DOI:** 10.1002/ece3.2590

**Published:** 2016-11-21

**Authors:** Duncan S. Procter, Joan E. Cottrell, Kevin Watts, Stuart W. A'Hara, Michael Hofreiter, Elva J. H. Robinson

**Affiliations:** ^1^ York Centre for Complex Systems Analysis & Department of BiologyUniversity of YorkYorkUK; ^2^ Centre for Exercise, Nutrition and Health SciencesSchool for Policy StudiesUniversity of BristolBristolUK; ^3^ Forest ResearchRoslinMidlothianUK; ^4^ Forest ResearchFarnhamSurreyUK; ^5^ Institut für Biochemie und BiologieUniversität PotsdamPotsdamGermany

**Keywords:** colony organization, eusociality, *Formica lugubris*, kin selection, polydomy, social organization

## Abstract

Eusociality is one of the most complex forms of social organization, characterized by cooperative and reproductive units termed colonies. Altruistic behavior of workers within colonies is explained by inclusive fitness, with indirect fitness benefits accrued by helping kin. Members of a social insect colony are expected to be more closely related to one another than they are to other conspecifics. In many social insects, the colony can extend to multiple socially connected but spatially separate nests (polydomy). Social connections, such as trails between nests, promote cooperation and resource exchange, and we predict that workers from socially connected nests will have higher internest relatedness than those from socially unconnected, and noncooperating, nests. We measure social connections, resource exchange, and internest genetic relatedness in the polydomous wood ant *Formica lugubris* to test whether (1) socially connected but spatially separate nests cooperate, and (2) high internest relatedness is the underlying driver of this cooperation. Our results show that socially connected nests exhibit movement of workers and resources, which suggests they do cooperate, whereas unconnected nests do not. However, we find no difference in internest genetic relatedness between socially connected and unconnected nest pairs, both show high kinship. Our results suggest that neighboring pairs of connected nests show a social and cooperative distinction, but no genetic distinction. We hypothesize that the loss of a social connection may initiate ecological divergence within colonies. Genetic divergence between neighboring nests may build up only later, as a consequence rather than a cause of colony separation.

## 1 Introduction

Understanding how and why animal societies are organized in the way they are has long been a focus of biological research. Eusocial societies, characterized by cooperative brood care, overlapping generations, and division of labor, are among the most complex forms of social organization. Eusociality is found throughout the animal kingdom, for example, in mammals and crustaceans (Duffy, Morrison, & Rios, 2000; Jarvis & Bennett, 1993), but is particularly widespread in insects (Inward, Vogler, & Eggleton, 2007; Johnson et al., 2013; Smith, Beattie, Kent, & Stow, 2009; Stern, 1998). In eusocial organisms, the colony is a fundamental unit of social organization; this reproductive and selective unit competes with other colonies within a population (Hölldobler & Wilson, 1990). Furthermore, the colony is also a cooperative unit; workers cooperate within colonies, collaboratively collecting resources and tending young, in order to produce the next generation.

Within a social insect colony, the workers do not themselves reproduce and are therefore behaving altruistically by helping the queens reproduce. This altruism can be explained by inclusive fitness theory, with indirect fitness benefits to the workers accrued via the enhanced reproduction of kin (Bourke, 2011; Hamilton, 1964). Positive relatedness between interacting organisms is required for the evolution of altruism, and as such, members of a social insect colony are expected to be more related to one another than they are to other individuals within the population. The positive effects of inclusive fitness can be further enhanced by ecological factors which result in higher benefits or lower costs of altruism (Bourke, 2011).

The traditional view of an ant colony is a single nest which contains a single queen and highly related workers. However, this view is increasingly being shown to be overly simplistic (Heinze, 2008). Ant colonies can contain multiple reproducing queens at any one time, a trait known as “polygyny” (e.g., Holzer, Chapuisat, Kremer, Finet, & Keller, 2006; Pedersen & Boomsma, 1999; Tsutsui & Case, 2001). In addition, the number of nests that comprise an ant colony can differ. Spatially discrete nests can operate functionally as a single colony, a situation termed polydomy (Debout, Schatz, Elias, & Mckey, 2007). Polydomy is found in widespread ecologically important species (Ellis & Robinson, 2014) and is a feature of some of the world's most damaging invasive species (e.g., *Pheidole megacephala* Fournier et al., 2012; *Linepithema humile* Gordon & Heller, 2014; *Anoplolepis gracilipes* Hoffmann, 2014). The suggested benefits of polydomy to the colony include the following: risk spreading (van Wilgenburg & Elgar, 2007), efficient resource acquisition and exploitation (Cook, Franks, & Robinson, 2013; Schmolke, 2009), escape from the limitations of a single nest site (Cao, 2013), or release from the inefficiency of a very large nest (Kramer, Scharf, & Foitzik, 2014; Robinson, 2014). All of these potential benefits of polydomy follow logically from the assumption that the colony is a cooperative unit, and this is reinforced by empirical evidence of cooperation in the form of resource exchange between nests (Buczkowski, 2012; Ellis, Franks, & Robinson, 2014; Ellis & Robinson, 2016; Gordon & Heller, 2014). Different methods for delineating ant colony boundaries do not always draw the same colony boundaries (Ellis, Franks & Robinson In Review; Ellis, Procter, Buckham‐Bonnett, Robinson In Review).

Polydomous colonies are defined as consisting of spatially separate nests linked by a social connection (Debout et al., 2007). Some ant species connect spatially separate nests with trails along which workers continually move back and forth, forming a clearly visible social connection (Ellis et al., 2014; Gordon & Heller, 2014; McIver, 1991). The strength of social connection between nests can be dramatic, with strong connections between nests involving hundreds of workers moving in either direction every minute (Skinner, 1980). Wood ants of the *Formica rufa* group, which includes *Formica lugubris*, use aboveground trail networks extensively (Rosengren, 1971); no examples of subterranean trail networks between nests are known in this group. Polydomous trail networks are structured to allow efficient transport of resources within the colony (Cook, Franks, & Robinson, 2014). In the wood ant *F.lugubris,* nests which, due to their network position, experience a higher flow of resources are more likely to grow, reproduce, and survive from year to year than those experiencing a lower resource flow (Ellis, Franks et al., In Review; Ellis, Procter et al., In Review). Polydomous trail networks therefore represent connections between cooperating nests (which we use as a shorthand to mean cooperating ant communities, each living within its own nest), sharing workers and resources, in line with the expectations of a social insect colony. In populations of *F. lugubris,* groups of nests connected by trails are often bordered by other nests to which they have no social connection, although the distance between unconnected nests can be similar to that between connected nests (D. Procter personal observation). Wood ant trails frequently persist in the same location for many years (Rosengren, 1971); furthermore, during mapping of trail networks of *F. lugubris* in the UK over 3 years, separate trail networks were never observed to become connected by trails (Ellis, Franks et al., In Review; Ellis, Procter et al., In Review), indicating stability of distinct networks of connected nests. *Formica lugubris* exhibits variation in dispersal strategies across its range, but in the UK, new nests are formed by budding, whereby one or several queens split off from the parent nest with a subset of the workers and form a new nest nearby (Hughes, 2006). Budding nest formation could result in neighboring nests with high genetic relatedness, allowing the formation of polydomous colonies.

In this study, we ask whether social connections between nest pairs correlate with genetic distinctions. To answer this question, we measure (1) worker movement, (2) carbohydrate resource exchange, and (3) genetic relatedness between neighboring nest pairs, which are either connected or unconnected by worker trails. The social connections characterized by worker and resource movement appear cooperative; therefore, we predict that they will be reflected by increased relatedness between socially connected nest pairs.

## 2 Methods

### 2.1 Study species and population

*Formica lugubris* Zetterstedt, 1838, is a member of the mound‐building red wood ants of the *Formica rufa* group, common across the temperate and boreal forests of Europe and Asia (Goropashnaya, Fedorov, Seifert, & Pamilo, 2004; Stockan & Robinson, 2016). The species exhibits variation in social structure throughout its range, but populations in Britain are polygynous and polydomous (Ellis & Robinson, 2014; Gyllenstrand & Seppä, 2003; Hughes, 2006). Red wood ants are ecologically dominant, a trait they share with many other polydomous species (Fournier et al., 2012; Gordon & Heller, 2014; Hoffmann, 2014). *Formica lugubris* forms strong trails both between neighboring nests and from their feeding grounds in aphid colonies in nearby trees to nests (Ellis et al., 2014; Sudd, 1983). The majority of the nutrient intake during the summer comes from honeydew from aphids (Rosengren & Sundström, 1991).

**Figure 1:**
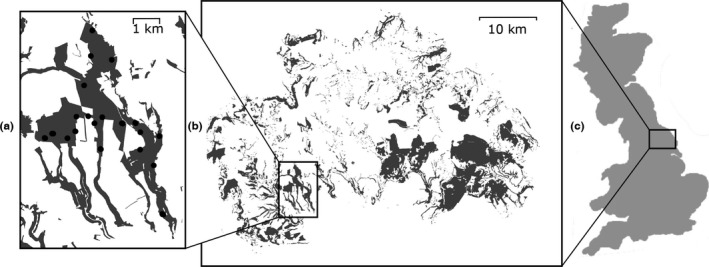
(a) The study *Formica lugubris* population: gray polygons are forest cover, and black circles are sampled triplet locations. Boxes denote (b) the population's location within the North York Moors National Park (again gray polygons are current forest cover) and (c) the location of the North York Moors within Britain

The study population is located in the southern half of the North York Moors National Park, in the northeast of England, UK (Long/Lat 54.289, −1.059, Figure 1). This landscape has undergone large increases in forest cover in the last 160 years, which has allowed concomitant expansions of the wood ant populations (Procter, Cottrell, Watts, & Robinson, 2015). The investigated population of *F. lugubris* contains approximately 3,000 nests, across an area of 10.4 km^2^ (Procter et al., 2015). This population was chosen for this study because prior knowledge of its extent and the location of nests allowed the selection of randomly distributed sampling points throughout the population, with sufficient spacing that any given polydomous colony, defined by social connections between spatially separate nests, did not span multiple sample points.

The forest is dominated by non‐native conifer plantations adjacent to sections of ancient broadleaf woodland. Commercial forests dominated by non‐native conifers represent a much more dynamic habitat than that provided by ancient woodland, due to relatively short harvest cycles, early canopy closure, and frequent management interventions. The more dynamic nature of commercial forests may cause faster nest turnover than in ancient woodland. Our sampling points cover both ancient woodland and commercial forestry plantations, allowing us to assess whether there was an effect of forest age on the internest genetic relatedness patterns we see within nest pairs. The age of the forest had no effect on these patterns; therefore, we present analyses only in the Appendix S1.

### 2.2 Mapping test triplets

**Figure 2:**
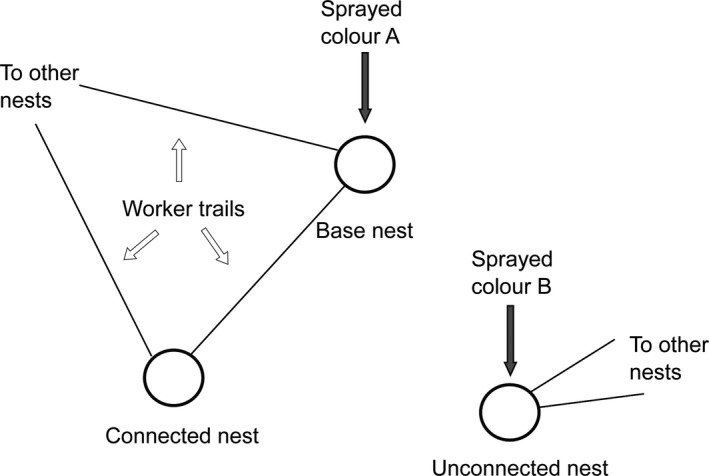
A schematic of the design for triplets used in this study: two nests connected by trails (arbitrarily termed “base” and “connected” nest) and a third nest (termed “unconnected”), a similar distance away but not connected by a trail. Spraying the base nest color A and the unconnected nest color B allows us to track worker movement from the base to connected nest, from the base to the unconnected nest, and from the unconnected nest to the base or connected nest. The unconnected nest was in some, but not all, cases connected to a separate nest network

The specific arrangement required for this study was a series of groups of three nests, where two nests in each triplet were connected by a trail of workers (arbitrarily termed the “base” and the “connected” nests) and the third nest was not connected directly or indirectly to either of the other two nests (termed the “unconnected” nest, Figure 2). In order to locate appropriate triplets, we began by randomly choosing 40 nests from previous survey data. Taking each randomly selected nest in turn, we mapped all nests to which the selected nest was connected by trails, either directly or indirectly (via one or more other nests), which resulted in a mapped network of nests connected by trails. We then searched the area immediately surrounding the mapped network of connected nests to find a nest close by that had no trail connection to any of the mapped nests (Figure 2 unconnected nest). If no appropriate unconnected nest was found, we moved on to the next randomly chosen nest and began again. We found the desired triplet arrangement on 24 of 40 occasions. The mapping took place in April and May 2014.

We attempted to find connected and unconnected nests for each triplet that were a similar distance from the base nest; however, overall, unconnected nests were significantly further away from the base nest (connected mean 8.9 m ± 8.3 SD, unconnected mean = 15.8 m ± 9.3 SD, paired *t*‐test, *t* = −4.59, *df* = 23, *p* < .001). To account for this difference in distance between the base nest and the connected or unconnected nest, the Euclidean distance, that is, straight line distance, between nests was included as a covariate in generalized linear mixed models during analysis.

It could have been possible that nest size explained the presence or absence of trails within triplets. For example, trails might only form between nests that are over or under a certain size. We therefore recorded nest volumes using the methods of Chen and Robinson (2013), which have been shown to correlate with worker populations, that is, the number of workers within the nest (Chen & Robinson, 2013), and tested for size effects on the presence of trails. None of the size effects was statistically significant (Appendix S2), so nest volumes were not included in further analyses.

### 2.3 Worker movement

We assessed worker movement between nests by mass‐marking ants on the nest surface with a single light application of spray paint (Painter's Touch Multipurpose Paint, Rust‐Oleum, Durham, blossom white and spa blue) on two nests in each of the 24 mapped triplets in June 2014. The paint brand was chosen because colors did not wear off, and the application of paint did not affect worker behavior (D. S. Procter, personal observation). The paint colors were chosen because they were both distinguishable from one another and clearly visible on the ants themselves. The ants on the base nest (Figure 2) were sprayed one color and those on the unconnected nest were sprayed a second color. The third nest within the triplet (“connected” in Figure 2) was not mass marked, because we could only find two paint colors that were both easily visible on the ants and distinguishable from one another. Nest surfaces were agitated before spraying, so that many workers from the interior came out onto the nest surface and were also marked. Colors were alternated between base and the unconnected nests in different triplets. We then returned to the sprayed triplet 1, 2, 3, 14, and 30 days after marking and counted the number of workers of each color on each of the three nests within the triplet by systematically scan‐sampling the surface of each nest. From this, we ascertained the relative level of worker movement from the base nest to the connected nest, the base nest to the unconnected nest, and the unconnected nest to the base nest (Figure 2). We tested whether the number of workers moving between nest pairs was significantly >0 using Wilcoxon rank tests in R (R Core Team, 2015).

### 2.4 Resource movement

We cannot assume that carbohydrate resource movement correlates with worker movement (Ellis, Franks et al., In Review; Ellis, Procter et al., In Review); therefore, we assessed internest resource movement independently of worker movement in a subset of 10 of the 24 mapped triplets in July 2014. We restricted the resource movement assessment to 10 of the triplets containing smaller nests, because in these smaller nests, we could be confident of detecting the marked food using a sample size of 100 workers per nest. The sampling limit was imposed by logistical constraints. Ants transfer sugar solution between colony workers via trophallaxis, the exchange of food mouth to mouth or mouth to anus (Hölldobler & Wilson, 1990). There is a large amount of ant activity around nests that does not occur along the internest trails; therefore, trophallaxis between workers of different nests could hypothetically be independent of the trails of workers between nests. Using a food bait approach, we assessed resource movement within the triplets by mixing sugar solution with Rabbit Immunoglobulin IgG (Sigma‐Aldrich) using the methods of Buczkowski and Bennet (2006). We focused on the transfer of resources from the base nest to others within the triplet using a single label. Sucrose solution (70%) in 1.5 ml volumes with 0.5 mg/ml IgG was placed in feeders made from inverted microcentrifuge tubes placed on top of the base nest of each triplet. We used 10 feeders per baited nest. Feeders were topped up 24 hr after initial placement on the nest surface. Samples of 100 workers per nest from each nest within the triplet were collected 48 hr after sugar solution was initially provided and sampled ants were placed in a chilled cool box. Upon arrival at the laboratory, the chilled workers were killed by placing them in the freezer at −20°C, where they were retained prior to analysis. Each sampled worker was assayed for IgG presence using an ELISA assay, carried out as follows: a 96‐well PCR plate was coated with 100 μl of anti‐rabbit IgG, diluted 1:500 in distilled water, and incubated at 4°C for 2 hr. Once incubation was complete, the primary antibody was discarded and 280 μl of 1% nonfat dry milk was added to each well as a blocker of any remaining nonspecific binding sites. After 30 min, the milk was discarded. Individual ant samples were homogenized in 200 μl phosphate‐buffered saline and vortexed, and 70 μl of each sample was added to a well in the prepared plate and incubated for 1 hr at room temperature. Samples were then discarded, and each well was washed three times with PBS Tween‐20 (0.05%) and then twice with phosphate‐buffered saline. Anti‐rabbit IgG conjugated to horseradish peroxidase diluted 1:1,000 in 1% nonfat dry milk was added to each well, after which the plate was incubated at room temperature for 1 hr. All wells then received the five washes described above before adding 50 μl of TMB (tetramethylbenzidine)–HRP (horseradish peroxidase) substrate (New England Biolabs) and incubated for 30 min at room temperature. Samples were analyzed on a BMG Labtech POLARstar OPTIMA microplate spectrophotometer set at an absorbance of 650 nm. Six negative controls which contained ants without IgG and six blanks which contained no ant sample were run on each plate. Individual wells were scored as positive if their absorbance value was more than three standard deviations higher than the mean of the negative controls (Buczkowski & Bennett, 2007). We analyzed differences in the number of workers testing positive for IgG between connected and unconnected nest pairs using a generalized linear mixed model (GLMM). The response variable was the number of workers testing positive for IgG, and we used a Poisson error structure. The explanatory variables were whether or not the nest pair was connected by a trail and the Euclidean distance between nests. The triplet the nest pair came from was included as a random effect. We used the glmer function in the lme4 package of R (Bates, Mächler, Bolker, & Walker, 2014).

### 2.5 Aggression

Aggression bioassays are a commonly used determinant of colony boundaries (e.g., Denis, Orivel, Hora, Chameron, & Fresneau, 2006; Garnas, Drummond, & Groden, 2007; Hölldobler, 1983; Kenne & Dejean, 1999), based on the assumption that workers will behave aggressively toward workers from neighboring colonies, but not their own colony mates. We conducted preliminary aggression studies in May 2014 (see Appendix S3 for details) on *F. lugubris* in our study landscape, but found that aggression levels were so low that aggression tests could not even distinguish behaviorally between populations that were separated by tens of kilometers, let alone neighboring colonies. We note that lack of aggression does not necessarily imply lack of colony‐mate recognition (Björkman‐Chiswell, van Wilgenburg, Thomas, Swearer, & Elgar, 2008; Holzer et al., 2006); however, we found no difference in antennation duration between tested workers from different locations (see Appendix S3 for details). We therefore decided not to deploy aggression bioassays in the full study, because they were unlikely to be informative.

### 2.6 Genetic distinctions between connected and unconnected nest pairs

We collected 10 workers per nest from each nest within 20 of the 24 triplets throughout the landscape in July 2014. We excluded four of the triplets used to assess worker movement, due to damage during the study period. All 10 triplets used to assess resource movement were included within the 20 sampled for genetic work. DNA was extracted using GeneJET Genomic DNA Purification kits following the manufacturer's instructions (Thermo Scientific). The sampled workers were each assessed for variation at the following 12 nuclear microsatellite loci: Fe7, Fe11, Fe13, Fe16, Fe17, Fe19, Fe21, Fe37, Fe38 (developed for *Formica exsecta* Gyllenstrand, Gertsch, & Pamilo, 2002), and Fl12, Fl20, and Fl21 (developed for *Formica paralugubris* Chapuisat, 1996; known as *Formica lugubris* type B at the time)*,* using the primers and PCR conditions specified in those papers. Each forward primer had a 5′—AGGTTTTCCCAGTCACGACGTT—3′ M13 sequence attached at the 5′ end for subsequent detection purposes. DNA was amplified in a total volume of 20 μl using the following reaction mixture: 1 μl DNA, 1X PCR buffer (Bioron, Germany), 5 μM of each primer (Integrated DNA Technologies), 0.2 mM of each dNTP (VWR International), 0.25 μM M13 oligo with either 700 or 800 nm fluorescent dye attached (Li‐Cor Biosciences), and 0.25U *Taq* DNA polymerase (Bioron). PCR products were diluted with formamide loading buffer and run on a Li‐Cor 4300 (Li‐Cor Biosciences, Lincoln, NE, USA). Allele sizes were scored by eye using a set of size standards for 700 and 800 nm wavelengths. Analyses based on genetic differentiation assume that loci are at Hardy–Weinberg equilibrium and there is no linkage disequilibrium between loci. Therefore, loci were tested for deviations from Hardy–Weinberg equilibrium and linkage disequilibrium within triplets in FSTAT 2.93 (Goudet, 1995).

We calculated pairwise genetic relatedness between all sampled workers in each triplet using the Triadic likelihood estimator of relatedness of Wang (2007) in the Coancestry 1.0.1.5 program (Wang, 2011), allowing for inbreeding in the population. Differences in internest genetic relatedness between workers from connected and unconnected nest pairs were analyzed as a generalized linear mixed model (GLMM) with binomial errors, because response values are constrained between 0 and 1. The response variable was the pairwise internest genetic relatedness between workers with explanatory variables being the nest pair on which the internest relatedness value was based (connected or unconnected) and the Euclidean distance between the pair of nests. Triplet identity was included as a random effect. The GLMM used the glmer function in the lme4 package of R (Bates et al., 2014).

We could not expect to see any differentiation between adjacent nests if there is no differentiation in the population as a whole. In order to confirm that there was differentiation within the population, we assessed isolation by distance for the 60 sampled nests within the population as a whole by measuring all pairwise *F*_ST_ scores between nests using the fst.pp function of the hierfstat package of R (Goudet, 2005). We then assessed whether there was a significant relationship between genetic distance (*F*_ST_/1 − *F*_ST_) and Euclidean distance between nests using a Mantel test with 9999 permutations, using the mantel.rtest function in the ade4 package of R (Chessel, Dufour, & Thiulouse, 2004). We also analyzed genetic differentiation between connected and unconnected nest pairs using hierarchical *F*‐statistics in the hierfstat package of R (Goudet, 2005). We separated the data into three hierarchical levels. Firstly, the differentiation among workers within nests, which we term *F*_Nest_, secondly, the differentiation between nests connected and unconnected by trails within triplets, termed *F*_Trail_, and lastly, the differentiation between triplets within the population, termed *F*_Trip_. *F*_Trail_ is the differentiation between those nests that share a social connection or do not, which is the value we are interested in this study. Statistical significance of the different hierarchical levels was determined by permutation tests with 1,000 permutations (Goudet, 2005).

Nonsignificant results indicate that there is no effect greater than that which is possible to detect given the experimental design employed. We conducted a power analysis in order to test the minimum level of difference in genetic relatedness we would be able to detect between connected and unconnected nest pairs. We simulated internest relatedness for the two treatments (pairs of connected and pairs of unconnected nests) based on characteristics of preliminary genetic data (mean relatedness 0.131, standard deviation = 0.055). We varied the difference in mean internest relatedness between connected and unconnected nest pairs between 0.001 and 0.1, at steps of 0.001. We simulated 1,000 variables per level of difference in treatments. Using 20 repeats, we achieved a power of 80% whenever the difference in relatedness between treatments was greater than 0.05; in other words, a significant difference (*p *< .05) between treatments was found in 80% of simulations. We were therefore confident that we could detect a significant difference in internest genetic relatedness between connected and unconnected nest pairs whenever the magnitude of the difference in relatedness was 0.05 or greater.

## 3 Results

### 3.1 Worker movement

**Figure 3:**
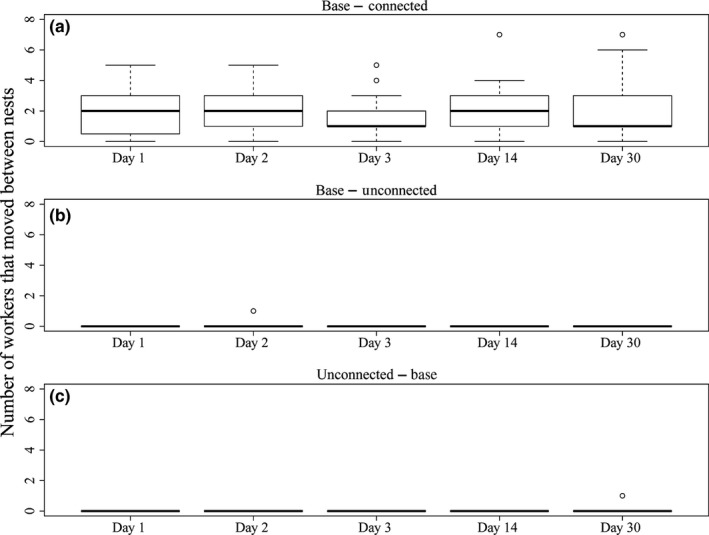
Number of workers that had moved from (a) the base nest to the connected nest, (b) the base nest to the unconnected nest, (c) the unconnected nest to the base nest, for each day of recounting for 24 triplets of nests. Boxes display 1st quartile, median, and 3rd quartile, whiskers extend to 1.5 IQ, and outliers are displayed as points

The number of ants detected to have moved between the base and connected nests in each triplet (Figure 2) was significantly greater than zero on all counting visits: 1, 2, 3, 14, and 30 days after paint marking (Wilcoxon rank test, *W* = 171–253, all *p *< .001, Figure 3a). In contrast, the number of ants that moved from the base nest to the unconnected nest did not significantly differ from zero on any counting visit (Wilcoxon rank test, *W* = 0–1, all *p *= 1, Figure 3b). Similarly, the number of ants moving from the unconnected nest to the base nest did not differ significantly from zero on any counting visit (Wilcoxon rank test, *W* = 0–3, *p *= .346–1, Figure 3c). Therefore, the presence of trails between nests does indicate a greater movement of workers, and the absence of trails does appear to mean a lack of social connection. The number of workers detected to have moved between connected nests on different days did not significantly differ (Kruskal–Wallis, *df* = 4, *χ*=1.46, *p *= .83, Figure 3).

### 3.2 Resource movement

**Figure 4:**
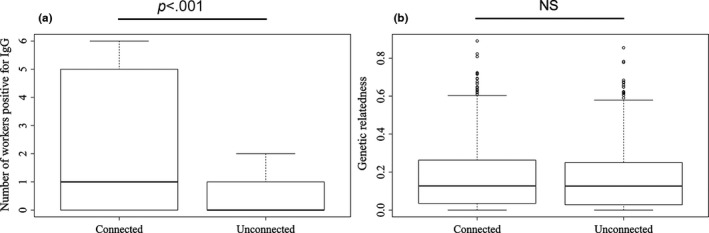
Comparisons between the connected and unconnected nest pair for (a) the number of workers testing positive for IgG (10 triplets) and (b) internest genetic relatedness (20 triplets). Boxes display 1st quartile, median, and 3rd quartile, whiskers extend to all points within 1.5 IQ, and outliers are displayed as points

After 48 hr of IgG marked sucrose being made available for ant feeding on the base nest, we detected a total of 279 of 3,000 collected workers positive for IgG. Of these, 252 were found on the baited base nest themselves, 22 on the connected nest, and only five on the unconnected nest. There were significantly more workers that tested positive for IgG on the connected nest than on the unconnected nest (GLMM, *df* = 1,4, *χ* = 9.34, *p *< .001, Figure 4a). There was no significant effect of Euclidean distance between nests on the number of workers testing positive for IgG (GLMM, *df* = 1,4, *χ* = 0.24, *p *= .62).

### 3.3 Genetic distinctions between connected and unconnected nest pairs

**Figure 5:**
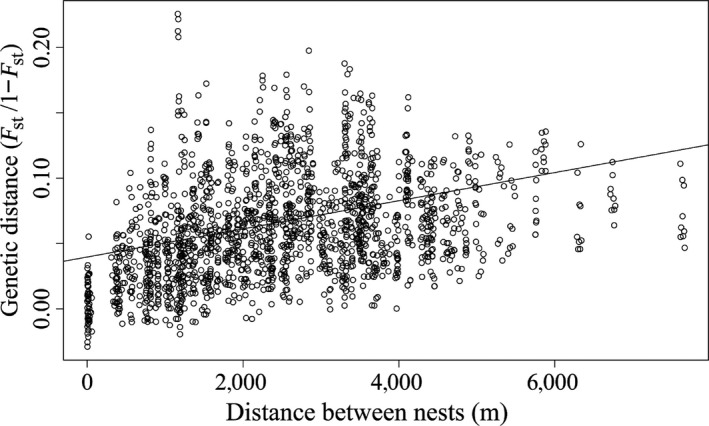
Genetic distance, measured by *F*_ST_/1 − *F*_ST_, against distance between all the sampled nest pairs in the population as a whole. The line displays a linear relationship between genetic distance and Euclidean distance between nests; significance was tested using a Mantel test

Diversity across the 12 microsatellite loci used ranged from low to high. Three of the loci displayed low variability (two‐three alleles, expected heterozygosity 0.16–0.51), with the remaining nine loci being more variable (4–19 alleles, expected heterozygosity 0.67–0.89). None of the loci showed significant deviations from Hardy–Weinberg equilibrium or significant linkage disequilibrium within samples, so all loci were retained for the analysis. The 60 nests making up the 20 triplets of nests in which workers were genotyped displayed significant isolation by distance, with genetic distance, measured by *F*_ST_/1 − *F*_ST_, increasing significantly as distance between nests increased (Mantel test, *r* = 0.36, *p *< .001, Figure 5).

Internest genetic relatedness between workers from connected nest pairs did not differ significantly from internest genetic relatedness between workers from unconnected nest pairs (connected pair mean = 0.17, unconnected mean = 0.16, GLMM, *df* = 1,3, *χ* = 0.122, *p *= .73, Figure 4b). There was no relationship between internest genetic relatedness and Euclidean distance within triplets (GLMM, *df* = 1,3, *χ* = 0.81, *p *= .36). The majority of differentiation was explained by the highest hierarchical level of organization of the data: the differentiation between different triplet groups, that is, the differentiation due to landscape patterns, which was significantly greater than zero (*F*_Trip_ = 0.055, *p *= .001). There was negligible differentiation between connected and unconnected pairs within triplets (*F*_Trail_ = 0.001), or within nests (*F*_Nest_ = 0.004), neither of which were significantly >0 (*F*_Trail_ *p *= .683, *F*_Nest_ *p *= .087). The negligible value of *F*_Trail_ supports the lack of difference in relatedness between connected and unconnected nest pairs, and high within triplet relatedness, that our relatedness analyses report.

## 4 Discussion

A social insect colony is expected to be a cooperative, reproductive, and selective unit, where members are more related to one another than to other members of the population. However, we have shown that workers from nests of *F. lugubris* that appear to cooperate are no more genetically related to one another than workers from nests that do not cooperate. Cooperation between ant nests involves the exchange of workers and resources. We have shown that both workers and resources move between connected nest pairs, whereas workers do not detectably move between unconnected nest pairs, and significantly fewer resources are exchanged. Nest pairs with an apparently cooperative connection neither differ in their internest genetic relatedness from unconnected nest pairs, nor do they display significant genetic differentiation from unconnected nests. The difference we observe in apparent cooperation therefore cannot be explained by a genetic difference.

Our results suggest that spatially separate nests in *F. lugubris* are cooperative units when connected by trails. Firstly, we have confirmed that trails between nests do constitute a social connection, because workers move between connected nest pairs but, more importantly, workers are not exchanged between unconnected nest pairs. Substantially, rarer movement between unconnected nests than connected nests is consistent with previous findings in a related species (O'Neill, 1988) and is expected if nests are solely cooperating within one colony. Secondly, we have shown that connected nests exchange significantly more resources than unconnected nests. Movement of resources between nests could be interpreted as either cooperation or stealing, but with stealing, we would expect competitive interactions. The strong social connections we observe, without aggression, suggest cooperation rather than competition. Existing evidence from other ant species suggests that new nests within polydomous colonies are placed near food sources (Holway & Case, 2000; Lanan, Dornhaus, & Bronstein, 2011). In *F. lugubris,* this does not appear to be the case; however, nests with workers that forage are more likely to survive than nonforaging nests (Ellis & Robinson, 2015). In *F. lugubris* colonies, workers appear to use nests they are connected to by trails as a foraging resource, which could be interpreted as a form of intraspecific kleptoparasitism (Ellis & Robinson, 2016). However, polydomous nest networks in wood ants and other species are structured to allow efficient transport of resources (Cook et al., 2014), suggesting there may be a colony‐level benefit to allowing, and even promoting, resource transfer between nests. In *F. lugubris,* a nest's position within the colony resource flow predicts nest survival (Ellis, Franks et al., In Review; Ellis, Procter et al., In Review), and nests within a network that do not forage are more likely to be abandoned (Ellis & Robinson, 2015). There is, therefore, an advantage to be connected to multiple nests, which should elicit competition between nests if connections are not cooperative. However, we find no aggression between neighbors in our population (see Appendix S3). Resource movement between spatially separate nests therefore suggests active cooperation between socially connected nests, as we predicted.

Our results clearly demonstrate that there is significantly higher resource transfer between nests connected by trails of workers that than between unconnected nests. However, in three of ten trials, we did see carbohydrate resource transfer between unconnected nests, albeit at a low level. The few workers that were found to be positive for IgG on the unconnected nest may have acquired resources from the baited nest via noncooperative means. The noncooperative acquisition of food could involve stealing from the baited nest or possibly inducing trophallaxis from workers from the baited nest. Trophallaxis is a standard method by which resources are transferred between workers of the same colony and is normally thought of as a sign of cooperation, but trophallaxis can also occur between species which do not cooperate (Bhatkar & Kloft, 1977). Under these circumstances, trophallaxis acts as a means of reducing interspecies aggression. Therefore, the exchange of resources seen in this study could be an activity that reduces aggression between colonies, analogous to reducing aggression between species. Resource movement can either correlate well with social connections (Heller, Ingram, & Gordon, 2008; VanWeelden, Bennett, & Buczkowski, 2015) or can operate at a different spatial scale (Buczkowski, 2012); therefore, the slight disparity between worker movement and resource movement in our results agrees with the literature: future studies should be cautious in assuming that social connections and resource movement are always closely correlated.

Workers themselves must also be considered resources for ant colonies, because they contribute to the production of the next generation. Our data support movement of workers between connected nests, which could be genuine worker exchange if the ants perform beneficial acts such as brood care or foraging for the recipient nests. While our current study does not investigate the behavior of the workers that move, worker movement may also be a form of resource exchange and arguably be more important than the exchange of carbohydrate, because carbohydrate maintains only the current generation of ants. Total resource exchange between nests is therefore a combination of worker exchange and exchange of food. Viewed in this way, the resource exchange between socially connected nests far exceeds the resource exchange between socially unconnected nests and represents a real cooperative distinction if the workers are behaving beneficially in the recipient nest.

We have shown that the apparent cooperative distinction we found is not reflected by a genetic distinction; however, we are not claiming that genetic factors are not important within ant colonies. The altruistic acts of workers within an ant colony are explained by inclusive fitness (Bourke, 2011; Hamilton, 1964), which includes both a benefit and cost term, as well as genetic relatedness. Genetic relatedness between the unconnected, noncooperative, nest pairs is remarkably high (mean = 0.16), indeed higher than is often observed within single nests of other ant species (e.g., in another *Formica* species as low as 0.01: Pamilo et al., 2005; and in other ant species 0.04: Goodisman & Ross, 1997; and 0.05 Pedersen & Boomsma, 1999). There is, therefore, no genetic reason why cooperative interactions should not occur. In *F. lugubris,* interactions between nests appear to be based on the movement of resources through the colony; ant nests that differ most in the amount of foraging that they perform are linked by stronger trails than those nests that had a more equal foraging effort (Ellis et al., 2014). In this study, we did not assess foraging in sufficient detail to determine the costs and benefits to each nest. If both nests within an unconnected pair forage sufficiently to support their worker force, then there may be no benefit to be gained from the presence of a trail between nests and therefore no reason to maintain a trail. Alternatively, because aphids are abundant in the vicinity of wood ant colonies, the exchange of carbohydrate between neighboring nests may incur only a tiny cost. With a small enough cost of resource exchange, there will be minimal evolutionary pressure to eliminate trails that are remnants of the nest formation event. Some trails may be lost by chance, while others are maintained, without a penalty to those that remain connected. We assume that the cost of the trail between nests is proportional to the length of that trail and accounts for trail length in our analyses. However, there may be other factors, such as desiccation or predation risk, that mean that trails between unconnected nest pairs are more costly than between connected nest pairs and preclude trail formation. We therefore suggest the distinction between connected and unconnected nest pairs is not caused by a genetic distinction, but by some unmeasured ecological or stochastic process.

Ants use cuticular hydrocarbons (CHCs) for nestmate recognition (Hölldobler & Wilson, 1990). The extent to which genetic and environmental patterns affect hydrocarbon profiles varies between ant species (Buczkowski & Silverman, 2006; van Zweden, Dreier, & D'Ettorre, 2009), but in wood ants, experimental separation has been shown to alter CHC profiles (Sorvari, Theodora, Turillazzi, Hakkarainen, & Sundström, 2008). It may therefore be that once a social connection has been lost for long enough for hydrocarbon profiles to diverge, genetically similar ants will no longer recognize one another as colony mates and the division becomes more permanent. Further studies may wish to assay CHC profiles alongside social connection methods to ascertain whether this is the driving factor.

The study landscape is dominated by commercial forests, which are both recently planted and highly dynamic in comparison with natural woodland. The addition of these commercial forests has benefitted the wood ants, allowing large population expansions (Procter et al., 2015). Due to these recent population expansions, we cannot expect the ant populations to be at equilibrium. It is possible that the recent range expansions of *F. lugubris* on the North York Moors have resulted in neighboring colonies exhibiting the high internest relatedness that we see. However, our sampled triplets were located in both ancient woodland and recently planted conifer plantations, and all showed the same lack of genetic distinction between connected and unconnected nest pairs (Appendix S1). We therefore think it is unlikely that the dynamic landscape will have masked any possible distinctions, but it would still be interesting to compare our results with a similar study in a less disturbed forest system.

The genetic patterns we report are based solely on nuclear DNA variation. Many ant species are known to exhibit sex‐biased dispersal, whereby males disperse larger distances than females. This results in differentiation in biparentally inherited nuclear genetic differentiation at a larger spatial scale than is seen for maternally inherited markers such as those located on mitochondrial DNA (e.g., Clémencet, Viginier, & Doums, 2005; Doums, Cabrera, & Peeters, 2002; Soare, Kumar, Naish, & O'Donnell, 2014). If there is a similar pattern of sex‐biased dispersal in this population, the division between connected and unconnected nest pairs may become exposed if mitochondrial DNA markers are used, because different matrilines within the connected and unconnected nest may be resolved. However, preliminary surveys of fragments of mitochondrial COI DNA showed only two haplotypes within this population, with variation never present within a sampled triplet (D. S. Procter unpublished data).

In the studied population, *F. lugubris* colonies reproduce by budding; this method of dispersal often results in strong spatial genetic structuring of populations, meaning that nests close to one another are more genetically similar irrespective of colony divisions (Sundström, Seppä, & Pamilo, 2005). Budding dispersal could therefore mean that all three of the nests in each of our triplets share common descent. Wood ant trails can be stable over long time periods (Rosengren, 1971). The trail structures within this population have not been mapped over multiple years, so we do not know how long the unconnected nests have been unconnected. However, in another *F. lugubris* population in the UK, trails have been mapped over multiple years: trail turnover does occur but new connections were not formed between separate trail networks, nor did trail networks separate and then reconnect (Ellis, Franks et al., In Review; Ellis, Procter et al., In Review). Therefore, there does appear to be a genuine separation between neighboring nest networks in *F. lugubris*. If unconnected nest pairs were connected, until recently, then our results indicate there has been insufficient time for genetic distinctions to build up between unconnected nests.

A social insect colony is expected to be a cooperative, reproductive, and selective unit, which should apply whether the colony occupies a single nest or multiple spatially separate nests. In a polydomous species, we suggest that there are cooperative divisions within genetically homogenous groupings. In some eusocial insects, social organization is to a degree controlled by environmental factors (Eickwort, Eickwort, Gordon, & Eickwort, 1996; Richards, 2000). Similarly, we suggest that it is ecology rather than genetics that is driving the polydomous nest organization that we observe here. Our findings support the polydomous colony as a cooperative entity, but not one that is genetically distinct from its neighbor. Our study also suggests that ecology plays a large role in determining social organization in this, and likely other, ant species.

## Data Accessibility

Microsatellite data, sampling locations, worker movement data, are archived in Dryad, DOI: 10.5061/dryad.4b072.

## Conflict of Interest

None declared.
