# Supplementary material for: Does cooperation mean kinship between spatially discrete ant nests?
Source: Ecol Evol. 2016 Nov 21;6(24):8846–56. doi: 10.1002/ece3.2590 (PMC5192893; doi:10.1002/ece3.2590)
Supplement: Supplementary file 1 [file ECE3-6-8846-s001.docx]

Supporting Information

**Appendix S1: The effect of forest age**

The forests within this population were planted at different time points, which might be expected to affect the inter-nest relatedness patterns we see. In the main analysis as presented in the paper, we did not take into account the age of forest and we concluded that there was no difference in inter-nest relatedness between connected and unconnected nest pairs (GLMM, df=1,3, Χ=0.12, P=0.73) If we include a variable separating triplets in ancient woodland (continuous since at least 1600) from the rest of the triplets in the models we still recover the same pattern in inter-nest relatedness: connected nest pairs do not show a significant difference in relatedness (GLMM, df=1,4, Χ=0.12, P=0.73). The age of forest when split into these two categories also has no significant effect on inter-nest genetic relatedness (GLMM, df=1,4, Χ<0.001, P=0.98, Fig. S1). If we incorporate a variable separating the triplets into three age classes (ancient woodland, forested since at least 1854, and forested more recently than 1854) then there is a significant effect of forest age on inter-nest genetic relatedness (GLMM, df=1,4, Χ=9.12, P=0.01, Fig. S2); however there is still no significant difference in inter-nest genetic relatedness between connected and unconnected nest pairs (GLMM, df=1,4, Χ=0.05, P=0.82). Therefore inter-nest genetic relatedness does vary with the age of forest in which the ants are located, but the pattern of inter-nest genetic relatedness between connected and unconnected nest pairs is unaffected. Further investigation of the effects of forest age on genetic patterns is beyond the scope of this paper.


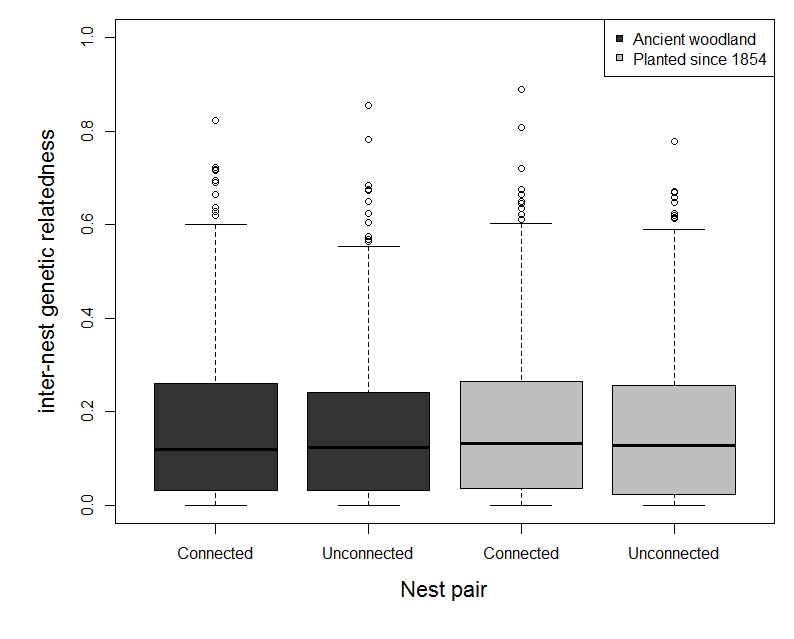


**Fig. S1**. Inter-nest genetic relatedness between connected and unconnected nest pairs (B-U), separated into two woodland categories: ancient woodland and woodland planted since 1854. Dark bars are ancient woodland and light bars planted since 1854.


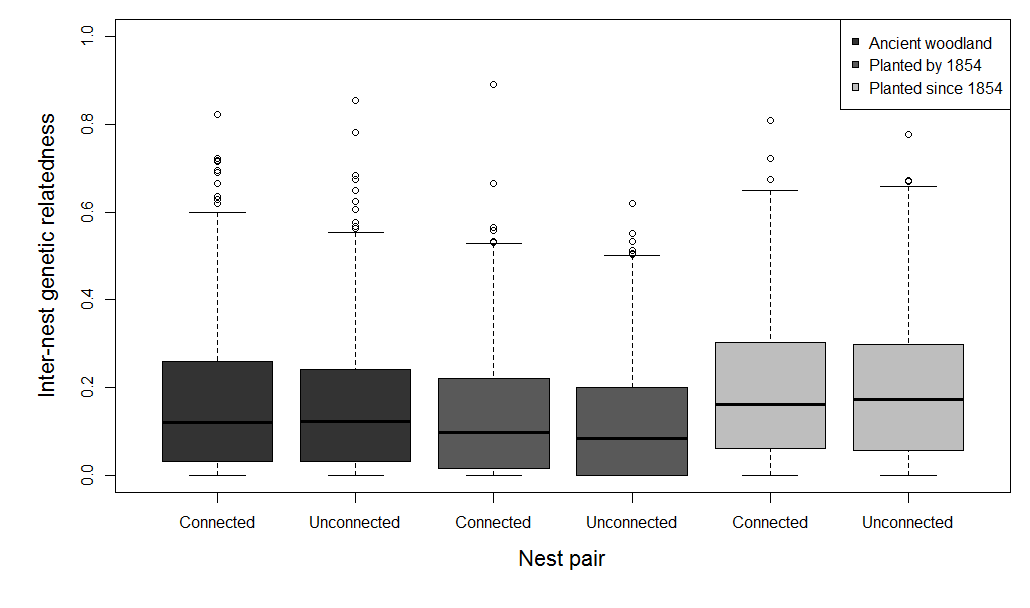


**Fig**. **S2.** Inter-nest genetic relatedness between connected and unconnected nest pairs separately for ancient forest (continuously present since before 1600, dark bars), forest planted by 1854 (mid-grey bars), and forest planted since 1854 (light grey).

**Appendix S2: The effect of nest size**

Hypothetically, nest size could be a predictor of trail presence, if, for example, nests under a certain size cannot maintain trails or if nests over a certain size have no need for them. We found no evidence of this. Mound volume, which correlates with worker population (Chen and Robinson, 2013), did not differ between the base, connected and unconnected nests (LMM, df=2, χ=0.95, P=0.62). There was no significant difference between the minimum volume of connected and unconnected nest pairs (LMM, df=1,3, χ=1.00,P=0.32). There was also no significant difference in the maximum nest volume of connected or unconnected nest pairs (LMM, df=1,3, χ=0.08, P=0.78). There was no significant variation in size difference between nests within the connected or unconnected nest pair (LMM, df=1,3, χ=0.007,P=0.93). There was also no significant difference in the combined volume of the connected nest pair and the unconnected nest pair (LMM, df=1,3, χ=1.61,P=0.21)

**Table S1.** Distances between different nest pairs within the triplet set up. For an explanation of the nest terminology see Fig. 2 in the paper

|  | Distance between nests (m) | | |
| --- | --- | --- | --- |
| **Triplet** | **Base-Connected** | **Base-Unconnected** | **Connected-Unconnected** |
| 1 | 13.62 | 11.34 | 14.02 |
| 2 | 2.46 | 29.21 | 31.66 |
| 3 | 2.95 | 7.71 | 7.15 |
| 4 | 18.58 | 29.5 | 47.83 |
| 5 | 3.56 | 5.05 | 4.99 |
| 6 | 26.62 | 35.2 | 57.34 |
| 7 | 3.3 | 9.96 | 22.85 |
| 8 | 6.37 | 7.8 | 13.41 |
| 9 | 7.63 | 14.47 | 16.02 |
| 10 | 2.26 | 11.93 | 14.09 |
| 11 | 4.23 | 8.33 | 12.5 |
| 12 | 7.44 | 8.12 | 15.45 |
| 13 | 26.92 | 32.32 | 59 |
| 14 | 4.3 | 13.9 | 10.82 |
| 15 | 3.85 | 19.63 | 16.76 |
| 16 | 2.72 | 5.68 | 8.37 |
| 17 | 1.32 | 10.76 | 11.89 |
| 18 | 13.94 | 26.42 | 26.98 |
| 19 | 22.97 | 17.88 | 28.98 |
| 20 | 3.16 | 11.77 | 14.15 |
| Mean | 8.9 | 15.8 | 21.7 |

**Appendix S3: Aggression bioassays**

We conducted preliminary aggression bioassays to assess whether they would be a useful tool for differentiating between cooperating nests and those that are in competition. We collected approximately 100 ants from each of five nests, three from one of the experimental triplets used in this study, one nest approximately 4km away from within the same continuous population and one approximately 15km away from a population separated from the study population by over 10km of wood ant-free habitat. Ants were taken from the field and housed with their nest-mates for 48 hours in the laboratory with ad libitum sucrose solution and protein.

To perform the assays we took a single ant from the base nest (see Fig. 2 in the paper) and placed it in a Petri dish with an ant from one of the five experimental nests. Tests were blinded by an independent assistant so the observer had no knowledge of where the second ant was from. Ants were allowed to acclimatise for one minute and then were observed for the subsequent five minutes. Interactions were scored on the following 0-3 scale and duration of interactions was measured:

0 - Ignore - Physical contact made but no antennation or aggression

1 - Touch - Antennation - Contact between antennae and other ant

2 - Avoid - Ant approaches and either upon contact or just before physical contact retreats from other ant

3 - Aggression - Biting, lunging with flared mandibles, positioning of abdomen ready to fire formic acid

A composite aggression score was then calculated as the mean score of all interactions. This was repeated until single ants from the base nest had been assayed with 10 of each of the five experimental nests. Each ant was used in a maximum of one trial. There was no difference in the composite aggression score between the different treatments (Kruskal-Wallis, df=4, Χ=2.97, *P*=0.56, Fig. S3). There was also no significant difference between antennation durations between treatments (Kruskal-Wallis, df=4, Χ=8.24, *P*=0.08, Fig. S4). Aggression bioassays were therefore not deemed to be a useful tool for this study


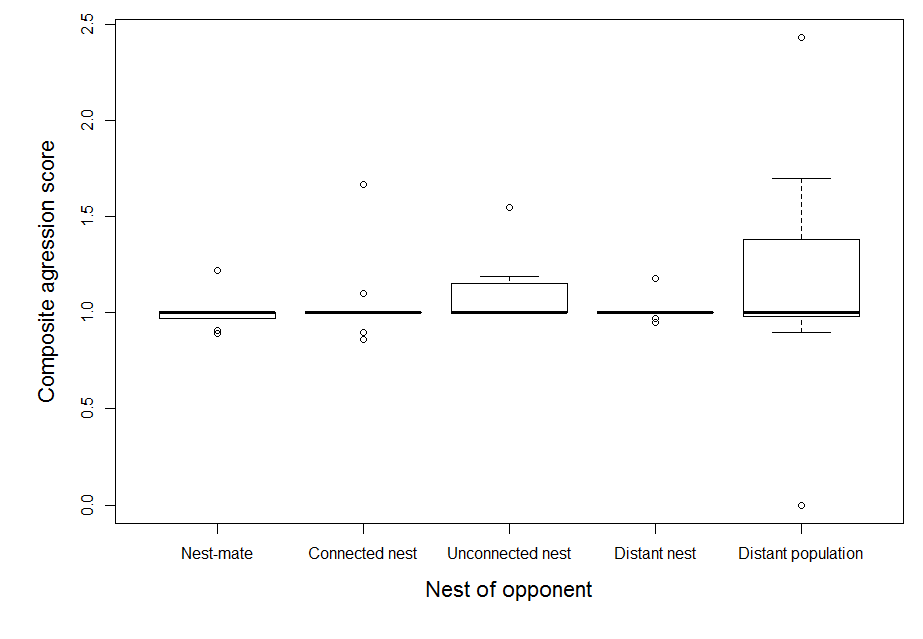


**Fig. S3.** The composite aggression score for 1vs 1 interactions between ants of the base nest (Fig. 2) and opponents from five different nests.


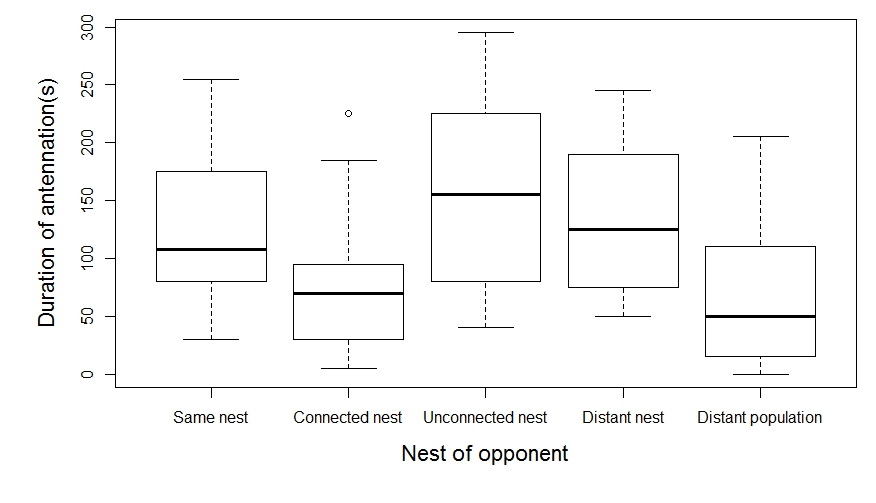


**Fig. S4.** The duration of antennations during 1vs 1 interactions between ants of the base nest (Fig. 2) and opponents from five different nests.
